# The neurocognitive disorder cohort RIFADE: Aims, methods, first results showing cognitive improvement in a subgroup

**DOI:** 10.1007/s00406-022-01516-3

**Published:** 2022-11-21

**Authors:** Bruno Baumann, Tim Lipka, Michaela Jänner, Milenko Kujovic

**Affiliations:** 1 Department of Psychiatry, University Hospital Münster, University of Münster, Münster, Germany; 2 Department of Psychiatry and Psychotherapy, Heinrich-Heine University Düsseldorf, Düsseldorf, Germany

**Keywords:** Neurocognitive disorder, Dementia, Prevention and control, Mild cognitive impairment, Risk factors

## Abstract

**Background:**

The NCD cohort study RIFADE (RIsk FActors of DEmentia) investigates the interaction of risk factors and neurocognitive disorders (NCDs) due to Alzheimer’s disease (NCD-AD) and NCD of vascular type (NCD-vascular). Retrospective recruitment referred to a period from 2007 to 2018 in a single centre. In addition to the baseline visit, follow-up visits took place at 3, 6, 12 months followed by yearly visits. Visit times varied in part depending on adherence. The study also comprises an EEG bank and a bank with cerebral MRI (c-MRI).

**Methods:**

Inclusion criteria were broad in order to cover a wide range of patterns of NCD. At baseline, patients underwent a large panel of assessments, e.g. including clinical history, diagnostic evaluation for NCD according to DSM-IV and NINDS AIREN criteria, a cognitive test battery including the DemTect, the clock drawing test and the Instrumental-Activities-of-Daily-Living-scale of Lawton and Brodie, EEG and c-MRI. At each follow-up visit, cognitive tests were repeated, in most cases also EEGs and in some cases c-MRIs. Numerous risk factors (RF) including vascular RF, atrial fibrillation, heart failure, sleep apnoea and lifestyle factors such as sedentary lifestyle, low cognitive style and smoking were evaluated for presence and for correction status at each visit, and modulation of uncorrected RF was initiated.

**Results:**

Overall, 126 subjects with a clinical diagnosis of NCD were included (52% female, mean age 71 ± 10.6 years (range 35e86)), number of follow-up visits per subject 2.9 ± 2.4, observation time per subject 3.4 ± 2.8 years). Of these, 55/28/17% presented with the clinical stages subjective cognitive decline (SCD)/mild cognitive impairment (MCI)/dementia (major NCD). Clinical diagnoses, retrospectively re-evaluated according to DSM-5, were 5/21/68/6% Alzheimer´s disease (NCD-AD)/vascular NCD (NCD-vascular) / mixed NCD (NCD-AD + NCD-vascular)/unspecified NCD. First longitudinal results revealed a mean DemTect score at baseline 12.6 ± 4.2 vs last visit 12.0 ± 4.8 (*p* = 0.08) and a clock drawing test score at baseline 1.9 ± 1.3 vs last visit 2.3 ± 1.5 (*p* < 0.0001). Of all subjects with MCI or major NCD (*n* = 57), 19 improved in the clinical stage from baseline to last visit (33.3%). Sixteen subjects progressed from SCD or MCI (*n* = 104) to major NCD (15.4%).

**Conclusion:**

The German NCD cohort RIFADE comprises patients with all clinical stages of NCD. A considerable subgroup improved in clinical stage. Further analysis is needed to answer the question of whether modulation of multiple risk factors provides a favourable effect on cognitive outcome in NCD.

## Introduction

In the last 16 years, global prevalence of dementia increased from 23.4 [1] to 55 million people [2]. Thus, former expectations of a doubling every 20 years have been exceeded, resulting in a rapid increase of social and individual burden. This is relevant, since recent research revealed an association of cognitive status and its course with quality of life [3] and mortality [4].

On the other hand, there is increasing evidence from hopeful data, suggesting that the up in prevalence rates may be slowing [5, 6]. An important impact on this outlook will be given by risk factors for dementia and their elimination. Recently, it was assumed that about one-third of new dementias could be prevented by control of modifiable risk factors [7].

Risk factors are increasingly becoming a focus of research on causes of changes in cognitive abilities, potentially resulting into dementia. Meanwhile, growing evidence exists for medical, psychological and environmental conditions, which turned out as drivers of cognitive decline [8, 9]. But there is still a lack of studies directly showing that the elimination of such risk factors could slow or even stop and reverse reduction of cognitive capacity.

One way to obtain direct evidence is to identify risk factors not only in terms of their presence status, i.e. to document whether a risk factor is present in the observed subject, but also to determine their correction status by answering the question of whether a risk factor has been eliminated. The correction status should ideally be estimated time-adapted to periods between repeated cognitive measurements in order to achieve a close insight into the temporal relationship of risk factors and cognitive course.

In a unified holistic approach, we propose that pathologies underlying dementia, such as Alzheimer pathology and its physiological consequences, should also be interpreted as risk factors, which interact with vascular and other risks. This view could overcome the restricted scope of previous therapeutic trials, which focused on a single factor, e.g. amyloid pathology, and often failed to be successful due to disregard of the multifactorial aetiology of neurocognitive disorders.

RIFADE, as a retrospective single-center observational study on the both most common types of dementia, Alzheimer´s disease and vascular dementia, is intended to be continued in a prospective design in order to replicate results and to capture new data after the advent of disease-modifying antidementive medications such as aducanumab [10]. The retrospective design, presented in this publication, should give first insights into the interaction and possible causal relationship of risk factors with Alzheimer´s disease, vascular neurocognitive disorder or the mixed form of both disorders. The cohort is registered on GermanCTR.de with identifier DRKS00027217.

## Materials and methods

### Study objectives

The primary aim of RIFADE is to study the effect of modifiable risk factors for dementia and their correction status on cognitive outcome in patients with neurocognitive disorder (NCD). As primary endpoint to define cognitive outcome, the DemTect [11] was chosen as a validated measure to categorize and predict outcome in NCD. The DemTect is used to differentiate mild cognitive impairment (MCI) from dementia [12]). It tests cognitive performance in terms of immediate as well as delayed recall of episodic memories, working memory and executive functions. It contains five subtests: word list, number transcoding, verbal fluency, digit span reverse and word list delayed recall, providing a maximum total score of 18. Secondary endpoints for outcome were the clock drawing test (CDT) [13] and the Instrumental-Activities-of-Daily-Living-scale of Lawton and Brodie (IADL) [14]. The CDT is a screening tool for dementia. It tests visuo-spatial function. During the test, probands are first asked to draw the face of a clock, then to add the hands, pointing to a predefined time (11:10). The accuracy of the drawing is then evaluated by correct order of the numbers and visual organization. The rating reaches from 1—perfect, meaning the correct time and no visual mistakes, to 6—no representation of a clock visible, meaning, for example, that words are written down instead of numbers. The IADL assesses daily functioning in terms of using the telephone, shopping, food preparation, housekeeping, laundry, mode of transportation, responsibility for own medications, and ability to handle finances. Each function is scored according to an algorithm, resulting in a score of 0 (no function) to 1 (good function). A maximum score of 8 can be achieved reflecting full abilities in daily functioning.

Secondary aims were:

- To collect data on functional alterations of the brain and their longitudinal time course in NCD by quantitative electroencephalography (QEEG)
- To evaluate the relationship between brain functional measures in QEEG and time course of NCD
- To evaluate the potential of longitudinal QEEG measures as a predictive marker in NCD
- To evaluate the potential of longitudinal QEEG measures as a prospective marker for dementia
- To evaluate whether structural biomarkers and white matter lesions in MRI interact with the presence and correction status of risk factors
- To investigate whether the pattern of morphological and functional indices, risk factors and diagnoses allows to reveal new disease phenotypes.

### Study design

RIFADE is a retrospective, observational single-centre cohort study. Patients were recruited from the Lower Rhine region in Germany near the Dutch border in an outpatient neurological ambulance (CNST Kalkar, Kalkar, Germany). This study centre pursues a scheme of a baseline visit and follow-up visits after 3, 6, 12 months followed by yearly visits. Similar to natural clinical settings, timing of follow-up visits was influenced by clinical acuity during the course of the NCD. RIFADE complies with the Declaration of Helsinki and Good Clinical Practice Guidelines and has been approved by The Ethics Committee at the Faculty of Medicine of Heinrich-Heine-University Düsseldorf.

### Study population

It was planned to include 120 patients following a chronological recruitment strategy. Inclusion criteria were restricted to the following categories of NCD [15]: major or mild neurocognitive disorder of Alzheimer type (NCD-AD), major or mild neurocognitive disorder of vascular type (NCD-vascular). Also patients with NCD of unclear aetiology (NCD-unclear) were included, if exclusion criteria were fulfilled.

Patients were enrolled, if the following inclusion criteria were fulfilled:

(i) Aged 35 years and older
(ii) Subjective cognitive complaints by patient, informant or due to clinical impression
(iii) Major or mild neurocognitive disorder due to Alzheimer disease (NCD-AD) according to DSM-5 (Diagnostic and Statistical Manual of Mental Disorders, Fifth Edition)
(iv) Vascular major or mild neurocognitive disorder according (NCD-vascular) to NINDS AIREN criteria [16]
(v) Neurocognitive disorder of unclear aetiology (NCD-unspecified)
(vi) First visit between January 1st 2007 and January 1st 2018 and if none of the following exclusion criteria were fulfilled:
(vii) Severe Parkinson’s disease
(viii) NCD due to fronto-temporal degeneration, Lewy-body-disease, Jakob–Creutzfeldt disease and other cerebral degenerative or inflammatory disorders except (iii) and (iv)
(ix) NCD due to extracerebral disorders
(x) Resident of a nursing home
(xi) Insufficient capacities in German language
(xii) Congenital deafness or blindness
(xiii) Apoplexy in the last 12 months
(xiv) Severe consuming disease with cachexia
(xv) Drug or alcohol addiction

Diagnoses were finally attributed in consensus conferences held by two board-certified and experienced psychiatrists and neurologists (BB, MK). Clinical records were evaluated if they refer to data between January 1 2007 and December 31 2020.

## Measurements

A broad panel of data was extracted from the clinical charts out of a certified medical documentation system (CompuGroup Medical M1Pro, Germany). Patients were only included, if they had at least 2 neurocognitive measurements.

Most measurements were taken already during the first visit in this cohort. Due to procedural algorithms and time delay between referral to and performance of measurements, echocardiography and polygraphy often occurred in later stages of treatment.

A number of comorbidities were systematically recorded by structured interviews: traumatic brain injury, surgical operations, severe infection disease with sepsis, severe stressors: loss of a child, job loss, divorce, loss of one or both parents or a sibling during childhood or youth, social isolation.

High priority was given to the record of risk factors for dementia. A focus was set on instantly modifiable established risk factors: arterial hypertension [26], diabetes mellitus [27, 28], hypoacusis [29], low cognitive style [30], sedentary life style [31] and atrial fibrillation [32]. A number of further potential risk factors were also recorded: deficit of vitamin B12 [33], obstructive sleep apnoea [34–39], heart failure [40, 41], vascular white matter lesions [42–44] and peripheral artery disease/coronary heart disease [45, 46]. Neuropathology of Alzheimer disease was evaluated as a further risk factor. Alzheimer pathology was recorded by the Scheltens scale and the ERICA score as biomarkers in cerebral MRI [47–49]. White matter lesions of vascular origin were recorded according to the Fazekas classification [50]. In line Table 1 with a Statement for Healthcare Professionals from the American Heart Association / American Stroke Association, the diagnosis of major and mild vascular neurocognitive disorder was separated into the categories “probable” or “possible” depending on the certainty of the relationship between the vascular disease and the onset of cognitive symptoms [51]. Under the condition of this relationship, probands with a Fazekas scoring of “1” for deep white matter hyperintensities in MR were also included into the vascular diagnostic spectrum in order to cover a broad range of vascular pathology. This also fits with the “Guideline-based approach to vascular impairment” proposed by Hachinski [52].

**Table 1:** Scheduled assessments and tests in the RIFADE cohort study

Assessment/Test	Details
Demography	Basic data, education, profession
Blood samples	Panel of samples (systemic inflammation, organ-specific markers, cholesterine, LDL, HDL, Lipoprotein a, HbA1c, Vit. B12)
Clinical history	Structured interview: comorbidities, familial history, medical support, cognitive symptoms in the last 3 years, neuropsychiatric inventory (NPI) [17]
Medication	Drugs currently used, interview for past medication
Anthropometric data	Weight, height
Risk factors for dementia	See Table 2
Polygraphy	Standard procedure [18]
Neurocognitive function and QEEG	
DemTect	Sensitive to early cognitive decrement [11]
Instrumental-activities-of-daily-living-scale (IADL)	Refers to daily functional abilities [13]
Clock drawing test	Tests visuo-spatial function [19]
Clinical dementia rating – Sum of boxes (CDR-SB)	Instrument for staging of dementia [20]
Quantitative EEG	Standard procedure [21]
Cardiology	
ECG at rest	Supine position, electronic recording and storage
Home blood pressure measurements (HBPM)	Continuous measurements over 5 days 3 × daily [22, 23]
Echocardiography	Adapted from the German Society for Cardiology
Carotid intima-media thickness (CIMT)	Optional; standard procedure
Exercise capacity and functioning	
Gait velocity	Measurement over 10 m [24]
Health-related questionnaires	
Depression (MADRAS)	Patient health questionnaire – depression [25]
Daily cognitive activity	Structured Interview (See Table 2)
Daily physical activity	Structured Interview (See Table 2)
Supply of cerebral MRI	If available (up to 5 years old). Semi-quantitative, standardized evaluation → imaging bank
MRI	Dedicated cerebral protocol

Another focus was set not only to record the presence status of a risk factor but also to address the question whether a risk factor was corrected or eliminated. Table 2 shows criteria for presence and correction status of risk factors investigated in this study. To study the influence of risk factors on cognitive outcome, neurocognitive time periods between 2 successive measurements of the primary outcome variable were established (NCT). Since the primary outcome variable was recorded as repeated measures, each patient exhibits at least 1 NCT. Each risk factor shown in Table 2 was evaluated for each NCT regarding 1.) presence status 2.) correction status. A time period of intervention (TI), resulting in a sufficient correction status, was recorded for each NCT. For example, in the case that no correction of a risk factor occurred, the final historical date of each NCT was recorded as start- *and* stop-time for TI, resulting in zero days of TI for this RF in this particular NCT. Thus, ratios could be calculated for TI / NCT as well as a predominant correction status, which was present in more than 50% of the NCT.

**Table 2:** Criteria for presence and correction status of risk factors

Risk factor	Criteria for presence status	Criteria for correction status
Arterial hypertension	Arterial hypertension was considered as present in case of 1.) pre-existing medication with an antihypertensive drug and/or 2.) a mean value > 140/90 mm Hg in at least 10 successive measurements during 5 days and/or 3.) anamnesis indicating existing arterial hypertension	Arterial hypertension was considered as corrected in case of 1.) regular intake of at least 1 antihypertensive drug and/or 2.) a mean value < 140/90 mm Hg in at least 10 successive measurements during 5 days
Hyperlipidemia	Hyperlipidaemia was considered as present in case of 1.) treatment with a statin or another antilipemic drug and/or 2.) reported history of a diagnosis of hyperlipidaemia and/or 3.) a serum level of cholesterine > 200 mg/dl and/or 4.) a serum level of triglyceride > 150 mg/dl	Hyperlipidaemia was considered as corrected in case of 1.) regular intake of a statine or another antilipemic drug and/or 2.) a serum level of LDL-cholesterine and triglyceride in the range of current ESC-guidelines [53]
Diabetes mellitus	Diabetes mellitus was considered as present in case of 1.) pre-existing medication with an antidiabetic drug and/or 2.) reported history of a diagnosis of diabetes and/or HbA1 serum level > 6.5%	Diabetes mellitus was considered as corrected in case of 1.) regular intake of an antidiabetic drug and/or 2.) regular intake of benfotiamine and 3.) a serum level of HbA1c < = 7.5%. Diabetes also was evaluated as corrected in case of only criterium 3 was fulfilled
Obstructive sleep apnea	Obstructive sleep apnoea was considered as present in case of 1.) Treatment with continuous positive airway pressure and/or 2.) a current outpatient-recording and/or polysomnography with a diagnosis of obstructive sleep apnoea	Obstructive sleep apnoea was considered as corrected in case of 1.) Treatment with continuous positive airway pressure and/or 2.) use of alternative therapy for obstructive sleep apnoea resulting in polysomnography with AHI < 10
Vitamin B12	Reduced availability of vitamin B12 was considered as present in case of serum levels < 400 ng/l	Reduced availability of vitamin B12 was considered as corrected in case of 1.) intramuscular/intravenuous injection of at least 1000 µg vitamin B12 per month or 2.) oral intake of at least 500 µg vitamin B12 per week
Atrial fibrillation	Atrial fibrillation was considered as present in case of 1.) reported history of a diagnosis of AF and/or 2.) current appearance in a 12-lead ECG for a period of > = 30 s	Atrial fibrillation was considered as corrected in case of 1.) successful attempts to restore continuous sinus rhythm and/or 2.) treatment by anticoagulation
Atherosclerotic disease/vascular brain lesions	Atherosclerotic disease with cerebral manifestation was considered as present in case of 1.) reported history of peripheral or coronary atherosclerotic disease and/or 2.) vascular lesions in cerebral MRI according to Fazekas criteria	Atherosclerotic disease with cerebral manifestation was considered as corrected in case of treatment with an antithrombotic drug
Probable Alzheimer pathology	A probable Alzheimer pathology was considered as present in case of 1.) a clinical diagnosis of neurocognitive disorder due to Alzheimer´s disease according to DSM-5 and 2.) hypotrophy of the temporal lobe according to the scale of Scheltens	Alzheimer-associated neurotransmitter dysregulations were considered as corrected in case of treatment with an approved antidementive drug such as cholinesterase inhibitors or memantine
Heart failure	Heart failure was considered as present in case of a reported history of a diagnosis of heart failure and/or a serum level of Brain Natriuretic Peptide > 150 pg/ml	Heart failure was considered as corrected in case of medication or other treatments improving heart failure according to current ESC-guidelines [54]
Sedentary life style	Sedentary Life Style was considered as present if according to anamnesis patient performed less than 1000 steps per day	Sedentary Life Style was considered as corrected if the patient took measures resulting in making > 1000 steps per day
Low cognitive style	Low cognitive style was considered as present if according to anamnesis the patient spent more than 5 h of missing cognitive activity during time awake	Low cognitive style was considered as corrected if the patient took measures resulting in less than 5 h of missing cognitive activity during time awake
Hearing loss	Hearing Loss was considered as present in case of 1.) a reported history of hearing loss and 2.) difficulties in bilateral communication without hearing aid	Hearing loss was considered as corrected in case of regular use of hearing aids
Nicotine	Nicotine abuse was considered present in case of a 1.) reported history of nicotine abuse and 2.) a current abuse of nicotine	Nicotine abuse was considered as corrected in case of stable abstinence from nicotine

### Quality control and assurance

In order to reduce errors during data capture, the following measures were taken:

Extensive plausibility checks and explanatory comments were included into the electronic CRFs (eCRFs). eCRFs were worked out by one co-author (TL) and supervised by another author (BB). The latter was the clinical investigator in the cohort. TL was regularly trained according to SOPs with regard to extraction of clinical data from clinical charts, evaluation of risk factors according to given criteria and data entry. EEG data were recorded and edited in a uniform manner according to SOPs. Semiquantitative evaluations of cerebral MRIs were performed by BB and MK according to standard criteria implemented in the used scoring algorithms.

### Statistical methods

Results presented in this article provide the descriptive analysis of the RIFADE data obtained at baseline. Additionally, the comparison between DemTect as primary outcome variable at baseline and at the following time point, and DemTect at baseline and at the last time point is described. Means and standard deviation are given for numeric variables. For categorical variables, absolute and relative frequencies are presented. Analysis was performed with SPSS 25. *N* = 126 patients were observed.

In addition, mixed effects repeated measurement models (MRMM) will be conducted including the different clinical stages and time as fixed effect. In order to reflect possible extra variability in repeated measurements originating from individual patients, variability of patients will be considered as random effect.

## Results

### Recruitment

**Fig. 1:**
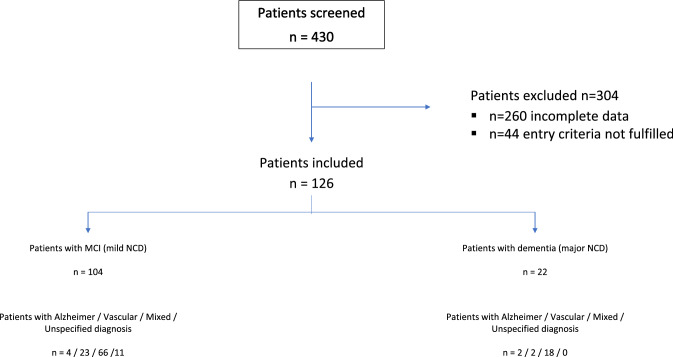
Patient Inclusion Flow Chart. *MCI* mild cognitive impairment, *NCD* neurocognitive disorder, *Alzheimer* due to Alzheimer´s disease, *Vascular* vascular NCD, *Mixed* NCD due to Alzheimer´s disease plus vascular NCD, *Unspecified diagnosis* unspecified NCD

A total of 126 patients were recruited from January 2007 to January 2018 in the single German study centre (cf. Figure 1). The end of observation was set for December 2020. Mean recruitment rate was 11 per year with a range from 4 (2007) to 18 (2011). Patients were referred by general practitioners or visited the centre on their own initiative or motivated by caregivers.

### Stages and classification of mild and major neurocognitive disorder

Classification of severity stages according to DSM-5 resulted in 104/22 patients with stages of mild / major neurocognitive disorder (NCD) corresponding to 82.5%/17.5% of the total study population. Based on the results of the DemTect at baseline, the group of mild NCD could be further divided in a group with subjective cognitive decline (SCD) with DemTect scores 13–18 and mild cognitive impairment (MCI) with DemTect scores of 9–12, resulting in 69/35 patients corresponding to 54.7%/27.8% of the study population (Table 3).

**Table 3:** Stages of neurocognitive disorder (NCD)

Demography	Missings	Total (*n* = 126, 100%)	SCD (*n* = 69, 54.7%)	MCI (*n* = 35, 27.8%)	Major NCD (*n* = 22, 17.5%)
Mean age (yrs, SD)	0	70.59 ± 10.61	67.56 ± 11.17	73.04 ± 9,5	76.19 ± 6.3
Female sex^1^ (*n*, %)	0	66 (52.4%)	34 (49.3%)	20 (57.1%)	12 (54.5%)
Education (yrs, SD)	0	9.26 ± 2.03	9.62 ± 2.54	9.18 ± 1.1	8.29 ± 0.7
Full and part-time employees (*n*, %)	0	16 (12.7%)	14 (20.3%)	2 (5.7%)	0

*yrs* years, *SD* standard deviation, *n* number of subjects, *SCD* subjective cognitive decline, *MCI* mild cognitive impairment, ^1^no intersex subject was recruited

According to the above-mentioned diagnostic classification of neurocognitive disorder, patients could be attributed to 86 mixed neurocognitive disorder (NCD-AD + NCD-vascular)/27 vascular NCD (NCD-vascular)/6 Alzheimer disease (NCD-AD) / 7 unspecified NCD (NCD-unspecified) corresponding to 68.3%/21.4%/4.8%/5.5% of the total study population. Among these 2 patients had unspecified diagnosis during the first NCT with mixed NCD in later NCT. Another 2 patients also had unspecified diagnosis during the first NCT with vascular NCD in later NCT.

### Baseline characteristics

The descriptive results of selected baseline characteristics are shown in Table 3. Patients were aged 35 to 86 years with a mean age of 71 years. There were less males (47.6%) than females (52.4%). Patients entering the study with a clinical stage of major NCD had less education (8.3 years) than the total cohort (9.3 years) and were older (76 years) and no one was employed any more, which contrasted with 14 patients with SCD, who were still employed (20.3%).

Classes of medications and comorbidities at baseline are given in Table 4. Number of risk factors per patient at baseline was 6.44** ± **2.20 with a range from 2 to 14.

**Table 4:** Prevalences of selected self-reported comorbidities and classes of medication at baseline

Comorbidities	*n*(%)	Medication	*n*(%)
Asthma	3 (2.38%)	Cardiovascular and metabolic medication	
Chronic bronchitis	3 (2.38%%)	Beta-Blocker	46 (36.51%)
Coronary artery disease	12 (9.52%)	Beta-Blocker + Diuretic	1 (0.79%)
Cardiac infarction	7 (5.56%)	ACE-Inhibitor	30 (23.81%)
Heart valve disease	0(0%)	ACE-Inhibitor + Diuretic	12 (9.52%)
Cardiac dysrhythmia	18 (14.26%)	Angiotensine receptor blocker (ARB)	15 (11.90%)
Stroke	19 (15.08%)	ARB + Diuretic	8 (6.35%)
Venous thrombosis	5 (3.97%)	Calcium channel blocker	25 (19.84%)
Gastritis	5 (3,97%)	Vasodilatators	2 (1.59%)
GE reflux disease	4 (3.17%)	Alpha2-Agonist	1 (0.79%)
Peptic ulcer	0 (0%)	Alpha-Blocker	2 (1.59%)
Diabetes with insulin	4 (3.17%)	Diuretic isolated	27 (21.43%)
Diabetes without insulin	12 (9.52%)	Potassium	0 (0%)
Gout	0 (0%)	Statin	39 (30.95%)
Tumor general	18 (14.26%)	Aspirin	35 (27.78%)
Arthrosis	3 (2.38%)	Other thrombocyte aggregation inhibitor	9 (7.14%)
Arthritis	5 (3.97%)	Anticoagulant	17 (13.49%)
Osteoporosis	6 (4.76%)	Antidiabetic oral	13 (10.32%)
Parkinson	10 (12.6%)	Insulin	4 (3.17%)
Restless legs	28(22,22%)		
Traumatic brain injury	5 (3.97%)	Neuropsychiatric related medication	
CNS Inflammatory disease	1 (0.79%)	L-Dopa	4 (3.17%)
Allergy overall	2 (1.59%)	Dopamin agonists	32 (25.40%)
Systemic inflammatory disease	0 (0%)	Antidementive	38 (30.16%)
Chronic Kidney disease	7 (5.56%)	Antidepressant	13 (10.32%)
		Other psychiatric medication	12(9.52%)
		Sum of any taken medication	385
		Medication per patient (mean)	3.06
Total *n*	126		126

### First results after study completion

A total of 366 neurocognitive time periods (NCT) defined by two successive DemTect measurements was observed in this cohort including 126 patients.

The number of NCT per patient was 2.9 ± 2.4. Mean observation time per patient was 3.4 ± 2.8 years.

The DemTect uses an age-dependent scoring algorithm to transform raw scores for age groups < 60 and ≥ 60 years. Since this algorithm might disturb analyses in observations of subjects passing the age border of 60 years, an age-independent scoring was used for 7 NCTs in which patients passed from age 59 to 60 years. Age-independent scoring was achieved in these NCTs by calculating both age-dependent transformed scores (the one for age < 60 and the one for age >  = 60 years) and averaging the both scores.

The change of DemTect scores from baseline to last visit did not correlate with time from baseline to last visit (*r*(126) = – 0.14, *p* = 0.13).

DemTect scores at baseline and at the last visit of each patient are shown in Table 5. Pre–post-comparisons revealed no significant effect.

**Table 5:** DemTect: baseline and post measurements

	DemTect baseline	DemTect last visit	F	Df	p
m	12.64	12.00			
SD	4.24	4.77			
n	126	126	3.148	1.125	0.078^1^

m mean*, SD* standard deviation, ^1^MANOVA pre-post-comparison

Similar comparisons for the clock drawing test provided a significant decline between baseline and last visit (*p* < 0.0001) (Table 6).

**Table 6:** Shulman’s clock test: baseline and post-measurements

	Clock test baseline	Clock test Last visit	Z	p
m	1.86	2.31		
SD	1.32	1.54		
n	125	125	– 3.539	< 0.0001*^2^

^*^ p < *0.05,* ^2^Wilcoxon test

**Fig. 2:**
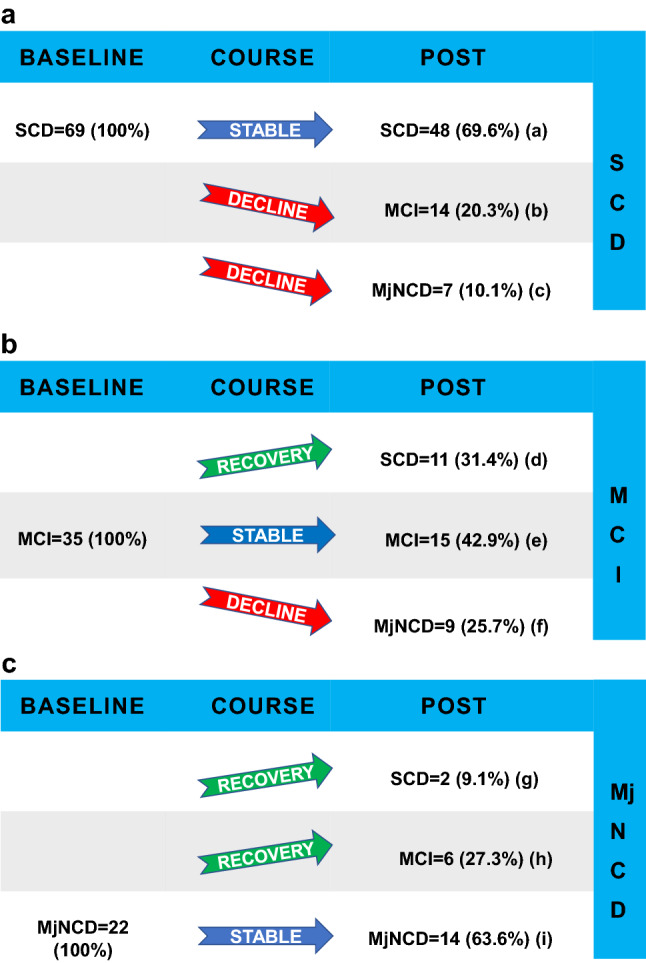
Transitions of cognitive stages from baseline to last visit. *SCD* subjective cognitive decline **A** *MCI* mild cognitive impairment **B** *MjNCD* major neurocognitive disorder **C**. Arrow up cognitive recovery, arrow horizontal cognitive stable, arrow down cognitive decline. Provided are numbers of subjects and percentages of each stage group. Times of observation (years): **a** 3.6 ± 2.9, **b** 4.3 ± 3.7, c 6.3 ± 2.5, **d** 3.1 ± 3.5, **e** 2.1 ± 1.1, **f** 4.8 ± 2.0, **g** 1.9 ± 1.0, **h** 2.1 ± 2.6, **i** 2.0 ± 1.1

Clinical stages SCD, MCI and major NCD at baseline and last observation are shown in Fig. 2. Of all patients with SCD or MCI (*n* = 104), 30 patients deteriorated in clinical stage (28.9%) in a mean time of 4.9 ± 3.0 years with 16 patients progressing to major NCD (15.4%) in 5.5 ± 2.3 years. Seventy-seven patients remained in the same clinical stage (61.1%) in a mean time of 3.0 ± 2.5 years; 19 patients of the group with MCI or major NCD at baseline (*n* = 57) improved in stage (33.3%) toward last observation in a mean time of 2.6 ± 3.0 years. Eight patients of the group with major NCD improved in stage (36.4%) with 6 patients reversing to MCI and 2 patients reversing to SCD.

Detailed analyses including the effect of risk factors and their correction will be presented in forthcoming separate papers.

## Discussion

The RIFADE cohort is a national NCD cohort studied by a comprehensive set of assessments and follow-up visits. Being a single-center study, it ensures a high level of uniform data collection. The cohort comprises the full spectrum of NCD severities, starting with the category SCD as an earliest stage of disease manifestation. The assessments include correction status of risk factors in a time-dependent manner allowing to analyse the potential cognitive effect of risk factor modulation. Uniform EEG records are available for the majority of patients enabling evaluation of QEEG measures and their longitudinal relationship with clinical course.

RIFADE shows a slight preponderance of female patients as expected from literature data [55, 56]. Generally sex differences could be studied given the similar portion of male patients, but such differentiations are statistically restricted by the small number of patients.

Whereas baseline characteristics are those typically seen in NCD, comparability of the longitudinal course to other NCD cohorts is made difficult by the intense therapeutic approach applied in the center where RIFADE patients were recruited. NCD cohorts generally reflect treatment as usual; thus, pooling of longitudinal data with other cohorts should be done with caution. On the other hand, clinicians are increasingly aware of the importance of risk factors, so that the data should be more comparable with future cohorts. This might be of importance since RIFADE is enrolled shortly before the advent of upcoming first disease modifiers for NCD-AD such as aducanumab [57].

The regional recruitment of RIFADE also limits the comparability with other cohorts. Therefore, RIFADE might not be fully representative of Germany as a whole due to different bio-psycho-social conditions throughout the lifespan as they might have occurred in West and East Germany. These differences could have an impact on the development and also on the adjustability of risk factors. In addition, regionally specific attitudes with different levels of adherence to medical treatment interventions may not be reflected in RIFADE.

Due to the fact that this is a monocentric study and a large number of exclusion criteria have to be considered, patient recruitment extended over a long period of about 10 years. This harbors the risk that comparability of patients with regard to risk factors could be limited in some aspects.

Treatment modalities may have changed and improved over the years. As a result, the corrective state of a risk factor could crystallize its effect on cognitive outcome all the more clearly. On the other hand, there were no decisive changes in the state of the art for their treatment in this period of time for any of the analysed risk factors. To date, all observed factors are mainly treated symptomatically.

Only a minority of RIFADE patients had major NCD. The most likely explanation is that many of these patients are handicapped to a degree that they were not able to visit the centre. Another reason could be the still widespread awareness, that dementia is hardly treatable in its final stages. It can be concluded that RIFADE patients with major NCD are “healthier” than common patients in this category. We do not consider underrepresentation as a major disadvantage. Probably clinical questions regarding patients with final stages of major NCD are better answered in specific studies focusing on behavioural rather than cognitive alterations.

The low portion of patients with pure NCD-AD in this cohort is remarkable and is in line with the view that the most common form of manifestation is NCD-AD in combination with vascular NCD [58]. Together with the mixed type including vascular NCD, NCD-AD accounts for 73% of RIFADE subjects. A similar number is often found in textbooks for the prevalence of AD in all-cause dementias without differentiation between pure AD and mixed forms.

Due to the retrospective design of RIFADE, patients show different times of observation. This makes a survival bias probable in that patients with an unfavourable course of the disease may have had an earlier loss of adherence. However, primary outcome of patients with only one follow-up visit was not significantly better than that of the group with additional follow-up measurements (*p* > 0.5). The most likely explanation is that patients lost adherence for both reasons of being satisfied and being disappointed with the success of treatment. Moreover, observation times showed no significant correlations with primary outcome, making a time bias less probable.

Regarding first longitudinal data, results of the DemTect as primary outcome measure deteriorated to a non-significant extent during total observation time. A clearer result was obtained by the clock drawing test, revealing a significant decrease of visual constructional capacities. Analyses using mixed linear models are planned to prove the influence of risk factors on cognitive abilities in NCD and to address the question of whether executive functions are less modifiable by modulation of the risk factors investigated in this study than other cognitive domains.

A key question of NCD cohorts undergoing an intensive therapy approach as given in RIFADE is whether there occur stable cognitive courses or even improvements over a period of years. The portion of stable patients remaining in their initial clinical stage (61.1%) and of patients with SCD or MCI progressing into major NCD (15.4%) is slightly better than in prior studies [59–61]. This result is surpassed by the occurrence of clinical improvements into better stages in 33.3% of patients with MCI or major NCD. This is in line with a recent report from an Australian cohort [62], showing transitions from MCI to a cognitive normal stage. In RIFADE, 36.4% of the patients with major NCD reversed to MCI or SCD. To our knowledge, this is the first study showing categorical improvements in subjects with major neurocognitive disorder to such an extent over a mean time of more than 2 years. Neither better education nor lower age can explain this effect. It is conceivable that increasing care for vascular and other risk factors in recent decades might have made possible such reversals from advanced clinical stages in NCD [63]. Analyses in forthcoming publications should show whether modulation of risk factors is responsible for favourable outcomes in a subgroup of RIFADE patients.

## Conclusion

 RIFADE is a NCD cohort focusing on a multitude of potential risk factors. Recruitment resulted in 126 patients of all NCD stages, for whom data of a large panel of assessments at baseline and repeated cognitive measurements were collected in high data quality. Detailed analyses of the effect of risk factors and their modulation on cognitive course are ongoing.
